# Randomized phase III trial of treatment duration for oral uracil and tegafur plus leucovorin as adjuvant chemotherapy for patients with stage IIB/III colon cancer: final results of JFMC33-0502

**DOI:** 10.1093/annonc/mdv358

**Published:** 2015-09-07

**Authors:** S. Sadahiro, T. Tsuchiya, K. Sasaki, K. Kondo, K. Katsumata, G. Nishimura, Y. Kakeji, H. Baba, S. Sato, K. Koda, Y. Yamaguchi, T. Morita, J. Matsuoka, H. Usuki, C. Hamada, S. Kodaira

**Affiliations:** 1 Department of Surgery, Tokai University, Isehara; 2 Department of Surgery, Sendai City Medical Center, Sendai; 3 Department of Surgery, Otaru Ekisaikai Hospital, Otaru; 4 Department of Surgery, National Hospital Organization Nagoya Medical Hospital, Nagoya; 5 Third Department of Surgery, Tokyo Medical University, Tokyo; 6 Department of Surgery, Japanese Red Cross Kanazawa Hospital, Ishikawa; 7 Division of Gastrointestinal Surgery, Department of Surgery, Graduate School of Medicine, Kobe University, Kobe; 8 Department of Gastroenterological Surgery, Kumamoto University, Kumamoto; 9 Department of Surgery, National Hospital Organization Himeji Medical Center, Himeji; 10 Department of Surgery, Teikyo University Chiba Medical Center, Ichihara; 11 Department of Clinical Oncology, Kawasaki Medical School, Kurashiki; 12 Department of Surgery, Aomori Prefectural Central Hospital, Aomori; 13 Department of Palliative Care, Okayama University Hospital, Okayama; 14 Department of Gastroenterological Surgery, Kagawa University, Kagawa; 15 Department of Management Science, Graduate School of Engineering, Tokyo University of Science, Tokyo; 16 Nerima General Hospital, Tokyo, Japan

**Keywords:** adjuvant chemotherapy, colon cancer, UFT/LV, treatment duration

## Abstract

**Background:**

While adjuvant chemotherapy is preferable for high-risk colon cancer, treatment duration is controversial. Oral uracil and tegafur (UFT)/leucovorin (LV) is widely used as a standard adjuvant chemotherapy for colon cancer in Japan. We conducted a phase III trial to investigate the optimal duration of adjuvant chemotherapy for stage IIB/III colon cancer.

**Patients and methods:**

Patients with curatively resected stage IIB/III colon cancer were eligible for enrollment in this trial. Patients were registered within 6 weeks after surgery and were randomly assigned to receive UFT/LV for 28 of 35 days for 6 months in the control group or for 5 consecutive days per week for 18 months in the study group. The primary end point was the disease-free survival (DFS), and the secondary end points were overall survival (OS) and safety.

**Result:**

A total of 1071 patients were registered from 233 centers. A statistically significant difference in DFS was not observed between the study group and the control group; the 5-year DFS was 69% in the study group and 69% in the control group. The 5-year OS was 85% in the study group and 85% in the control group.

**Conclusion:**

Eighteen-month treatment with UFT/LV did not improve DFS or OS compared with 6-month UFT/LV treatment in patients with stage IIB/III colon cancer. The important finding from this study is that not 18 months but 6 months of treatment is enough for postoperative UFT/LV for stage IIB/III colon cancer.

**Clinical trial number:**

UMIN-CTR C000000245.

## Abstract (precis)

While adjuvant chemotherapy is preferable for colon cancer, treatment duration is controversial. This phase III trial is investigated optimal duration of adjuvant chemotherapy for Stage IIB/III colon cancer. Eighteen-month treatment with UFT/LV did not improve DFS compared with 6-month UFT/LV treatment. This study suggests that 6 months treatment duration is enough for Stage IIB/III colon cancer.

## introduction

Colon cancer affects as many as 1.2 million people worldwide and more than 100 000 people in Japan every year [1]. Surgery is the mainstay of treatment of colon cancer, and postoperative adjuvant chemotherapy may be used to prevent postoperative recurrence, although not all patients benefit from this treatment. The benefit of adjuvant chemotherapy for stage II colon cancer is small [2], and routine use for all patients with stage II is not supported. For high-risk stage II colon cancer, however, adjuvant chemotherapy is considered a reasonable approach [3].

Adjuvant chemotherapy is standard treatment of stage III colon cancer and has also been recommended for the management of high-risk stage II colon cancer [3]. To date, only 5-fluorouracil (5-FU) alone and 5-FU plus oxaliplatin have been shown to be useful as postoperative adjuvant chemotherapy regimens for colon cancer [4–9].

To further improve the outcome of postoperative adjuvant chemotherapy for colon cancer, it is necessary to increase dose intensity, and extension of the treatment duration should be considered because 6 months may be insufficient. In studies comparing the usefulness of intravenous 5-FU//leucovorin (LV), oral capecitabine, oral uracil and tegafur (UFT)/LV, and oxaliplatin-based regimen as adjuvant chemotherapy, the duration of treatment was 6 months. Since the rate of recurrence reaches a peak between 1 and 2 years after surgery [10, 11], treatment duration for more than 1 year may suppress recurrences in a larger number of patients. To date, no trial has compared the effectiveness of 6-month to ≥1-year treatment with 5-FU/LV, and the optimum duration of treatment of each regimen remains unknown. Retrospective studies have suggested that long-term FU-based chemotherapy leads to favorable survival outcomes [12]. For long-term treatment, it is necessary to choose therapeutic regimens that are less likely to cause adverse effects and are superior in durability.

A 5-day treatment +2-day rest regimen of UFT that lasts 12 months was shown to be useful in the NSAS-CC study [13]. Sadahiro et al. reported that a 5-day treatment +2-day rest regimen of UFT was superior with respect to patient compliance and tolerability, and the concentration of 5-FU was maintained at relatively high levels in tumors during the 2-day rest period [14]. UFT/LV is an oral therapy that was shown to be noninferior to intravenous 5-FU/LV in JCOG0205 [15]. The present phase III study compared 6-month and 18-month durations using UFT/LV to determine whether long-term adjuvant chemotherapy is beneficial for patients with high-risk stage II and stage III colon cancer.

## methods

### patients

Patients were eligible if they had undergone radical resection of colorectal cancer with extended (D2 or more) lymph-node dissection and had pathologically complete resection of stage IIB (T4, N0, M0) or stage III cancer of the colon or rectosigmoid according to the tumor–node–metastasis classification of the International Union against Cancer, sixth edition. Other eligibility criteria included age 20–75 years, an Eastern Cooperative Oncology Group performance status (PS) of 0 or 1, no previous chemotherapy or radiotherapy, could take drugs orally, adequate organ function, and ability to start postoperative adjuvant chemotherapy within 6 weeks after surgery.

The study was approved by the institutional review board or ethics committee at each participating hospital and was conducted in accordance with the provisions of the Declaration of Helsinki and Ethical Guidelines for Clinical Research (overall revision dated 28 December 2004). All patients provided written informed consent.

### randomization and masking

The treatment assignments were randomized at the registration office in the Japanese Foundation for Multidisciplinary Treatment of cancer (JFMC) data center. Patients were randomly assigned (1:1) to receive UFT/LV either for 6 months (control group) or for 18 months (study group).

A minimization method was used to balance assignments according to the following stratifying factors: TNM T category (T1–2, T3, T4), N category (N0, N1, N2), surgical procedure (laparoscopic surgery, open surgery), and institution. The study investigators and patients were not blinded to the treatment assignments.

A data and safety monitoring board reviewed safety data during the study.

The study was funded and conducted by JFMC. No commercial support was involved in the study.

### treatment

The control group received UFT (300 mg/m^2^/day as tegafur) orally in three divided doses per day (every ∼8 h), avoiding 1 h before and after meals. LV (75 mg/day) was given orally in three divided doses per day at the same times as UFT. Drugs were administered for 28 consecutive days, followed by a 7-day rest (consecutive-day treatment), and this was defined as one course of treatment. Five courses of treatment (6 months) were administered. The study group received UFT plus LV at the same dose level as the control group. The drugs were administered orally for 5 consecutive days, followed by a 2-day rest. Five weeks of this regimen (5 days of treatment followed by a 2-day rest on Saturday and Sunday) were defined as one course of treatment, and 15 courses (18 months) were administered. After completing the scheduled treatment, patients were followed up with no further treatment until confirmation of metastasis or recurrence. The assigned treatment was started within 6 weeks after surgery. Additional details, i.e. dose modifications, have been previously reported [16].

### follow-up

During protocol treatment, clinical findings and laboratory data were evaluated every 2 weeks during the first two courses of treatment and then on the starting day of each subsequent course.

After completion of the protocol treatment, patients were followed-up according to a predefined surveillance schedule until recurrence or death was confirmed for 5 years after surgery. Recurrence was assessed based on CT scans. These tests were carried out every 4 months during the first 2 years after surgery and once every 6 months from the third year onward.

### statistical analysis

The primary end point was disease-free survival (DFS) and secondary end points were overall survival (OS) and safety. In this study, to achieve a power of 80% and an *α* of 0.05 (two-sided) by the Schoenfeld and Richter method, we required 398 patients for each group, 796 in total, with 2 years of accrual and 5 years of follow-up, assuming a 5-year DFS rate of 75% in the control group and hazard ratio (HR) of 0.667 for the investigational group over the control group. Therefore, the target sample size was set at 840 patients, assuming that the percentage of ineligible patients would be ∼5%.

DFS was defined as the period starting from the day of enrollment and ending on the day of recurrence; the day a cancerous, non-recurrent lesion (either synchronous or metachronous) was detected for the first time after the day of enrollment; or the day of death from any cause, whichever was earlier. The superiority in DFS was verified in all eligible patients using a log-rank test stratified by assignment factors (excluding study site; two-sided, significance level of 5%). For time-to-event analyses, the Kaplan–Meier method was used to estimate the survival rate for each group at each time point, and Greenwood's formula was used to calculate the confidence interval (CI). In addition, the Cox proportional hazards model was used to estimate the HR between treatment groups.

OS was defined as the period from the day of enrollment to the day of death from any cause. OS was analyzed in the same manner as for DFS. Safety was evaluated using Common Terminology Criteria for Adverse Events v3.0, by compiling adverse events in the treatment group and comparing standard and investigational treatment groups in terms of the incidence of adverse events. Data were analyzed using SAS version 9.2 software (SAS Institute, Cary, NC, USA).

This trial is registered with UMIN-CTR [http://www.umin.ac.jp/ctr/] (C000000245).

## results

### study population

A total of 1071 patients were enrolled at 233 hospitals in Japan between October 2005 and September 2007. Although the original target sample size was 840, the recruitment was continued throughout the scheduled registration period for 2 years to implement results of deliberation by the JFMC Clinical Trial Committee and finally 1071 patients were registered for the study.

**Figure 1.**
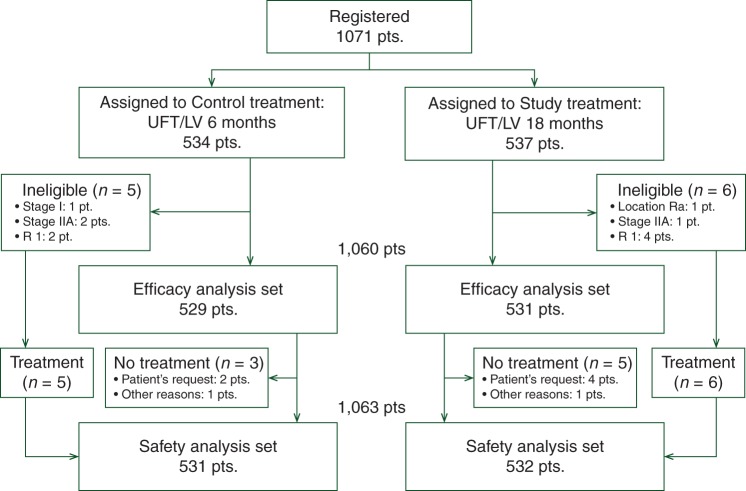
Consort diagram.

After excluding eight cases for reasons shown in Figure 1, 1063 patients were included in the safety analysis set and the safety results have been reported previously [16]. A total of 1050 patients (control group, 529 patients; study group, 531 patients) were included in the efficacy analysis set after excluding 11 ineligible cases. Patient demographics were well balanced in the two groups (Table 1).

**Table 1.** Patient characteristics

	Control		Study		Total	
	*N* = 534	%	*N* = 537	%	*N* = 1071	%
Sex						
Male	294	55.1	264	49.2	558	52.1
Female	240	44.9	273	50.8	513	47.9
Age (years)						
≤50	51	9.6	51	9.5	102	9.5
51–60	140	26.2	154	28.7	294	27.5
61–70	231	43.3	228	42.5	459	42.9
71–80	112	21	104	19.4	216	20.2
Median	64 (23–75)		64 (24–75)		64 (23–75)	
PS						
0	503	94.2	517	96.3	1020	95.2
1	31	5.8	20	3.7	51	4.8
Tumor location						
Right colon (C, A, T)	199	37.3	218	40.6	417	39
Left colon (D, S)	221	41.4	211	39.3	432	40.3
Rs	114	21.3	108	20.1	222	20.7
Operative procedure						
Laparoscopic	109	20.4	110	20.5	219	20.4
Laparotomy	425	79.6	427	79.5	852	79.6
Histological type						
Well	187	35	190	35.4	377	35.2
Mod	308	57.7	307	57.2	615	57.4
Poor	19	3.6	20	3.7	39	3.6
Muc	20	3.7	18	3.4	38	3.5
Sig	0	0	2	0.4	2	0.2
T (TNM 6th)						
T1	16	3	16	3	32	3
T2	51	9.6	45	8.4	96	9
T3	283	53	272	50.7	555	51.8
T4	184	34.5	204	38	388	36.2
N (TNM 6th)						
N0	69	12.9	75	14	144	13.4
N1	347	65	352	65.5	699	65.3
N2	118	22.1	110	20.5	228	21.3
Stage (TNM 6th)						
I	1	0.2	0	0	1	0.1
IIA	2	0.4	1	0.2	3	0.3
IIB	66	12.4	74	13.8	140	13.1
IIIA	59	11	57	10.6	116	10.8
IIIB	288	53.9	295	54.9	583	54.4
IIIC	118	22.1	110	20.5	228	21.3
Extent of LN dissection						
D2	147	27.5	136	25.3	283	26.4
D3	387	72.5	391	72.8	778	72.6
No. of LN examined						
<12	165	30.9	151	28.1	316	29.5
≥12	369	69.1	386	71.9	755	70.5

In the control group, 12.4% of patients were stage IIB and 87.0% were stage III. In the study group, 13.8% were stage IIB and 86.0% were stage III. A total of 11 patients did not meet the eligibility criteria related to baseline characteristics. In the control group, three patients were stage I or IIA and the resection of the primary tumor was incomplete in two patients. In the study group, the resection of the primary tumor was incomplete in four patients, primary tumor was located in the middle rectum in one patient, and one patient was stage IIA.

### disease-free survival

**Figure 2.**
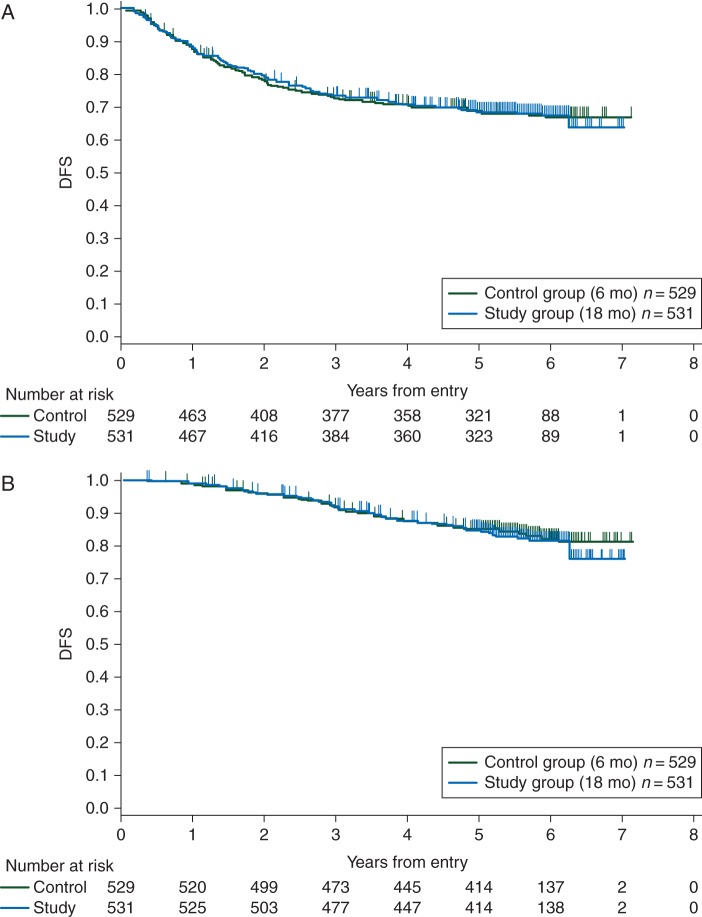
(A) Disease-free survival: The hazard ratio in the study group when compared with the control group was 1.00 (95% confidence interval 0.80–1.24, stratified log-rank test, *P* = 0.98). (B) Overall survival: The hazard ratio in the study group when compared with the control group was 1.05 (95% confidence interval 0.78–1.42, stratified log-rank test, *P* = 0.73).

DFS was analyzed based on 334 events (31.5%); i.e. 167 events (31.6%) in the control group and 167 events (31.5%) in the study group. Five-year survival was 68.8% (95% CI 64.6–72.6) in the control group and 68.9% (95% CI 64.7–72.7) in the study group. The study group did not show statistical superiority over the control group (HR = 1.00; 95% CI 0.80–1.24; stratified log-rank test, *P* = 0.98; Figure 2A). The first relapse was observed in 135 patients (25.5%) in the control group and 132 patients (24.9%) in the study group. In the control and study groups, the major sites of recurrence were as follows: local recurrence in 12 (8.9%) and 15 patients (11.4%), the liver in 55 (40.7%) and 45 patients (34.1%), lungs in 33 (24.4%) and 28 patients (21.2%), lymph nodes in 14 (10.4%) and 16 patients (12.1%), and the peritoneum in 9 (6.7%) and 22 patients (16.7%), respectively. Year-to-year changes in DFS in this study (supplementary Table S1, available at *Annals of Oncology* online) show that recurrence occurred most frequently between 1 and 2 years in both groups.

### overall survival

OS was analyzed based on 177 events (16.7%); i.e. 86 events (16.3%) in the control group and 91 events (17.1%) in the study group. Five-year survival was 84.9% (95% CI 81.5–87.7) in the control group and 84.5% (95% CI 81.1–87.4) in the study group. The HR for the study group over the control group was 1.05 (95% CI 0.78–1.42; stratified log-rank test, *P* = 0.73; Figure 2B).

### chemotherapy and safety

Six-month treatment completion was 74.0% in the control group and 75.8% in the study group, and 18-month treatment completion was 56.0% in the study group. Details of the safety analysis have been reported previously [16].

In brief, the overall incidence of adverse events was 75.3% in the control group and 77.6% in the study group. The incidence of grade 3 or higher adverse events was low in both groups. Diarrhea was the only grade 3 or higher adverse event with an incidence of >5%, and occurred in 7.2 and 2.4% in the control and study groups, respectively. There was no significant difference in safety between the groups, and treatment was tolerated in both groups.

At the completion of five courses of treatment, adverse events of any grade had been reported in 69.2% of patients in the study group. During the first 6 months, the incidence of subjective adverse events was significantly lower in the study group.

## discussion

To further improve the outcome of postoperative adjuvant chemotherapy for colon cancer, we claim that it is necessary to increase the dose intensity and consider extending treatment duration. In Japan, oxaliplatin as adjuvant therapy became available in August 2009; i.e., oxaliplatin was not available at the initiation of this study. Moreover, for long-term treatment ≥6 months, convenience of use and safety are important factors for patients, and 5-FU is preferred due to its convenience and ease of use in routine practice. Therefore, we decided to use UFT/LV, for which the efficacy and safety have been shown to be equivalent to 5-FU/LV in 6-month treatment regimens in the NSABP C06 study, as the investigational drug. None of the clinical studies conducted to date have led to the conclusion that the optimum duration of treatment with fluorinated pyrimidines as postoperative adjuvant chemotherapy for colon cancer is 6 months. Therefore, it is considered necessary extending the treatment duration to 1–2 years.

In clinical trials carried out in Japan, 1 year or 2 years of postoperative adjuvant chemotherapy with UFT alone, the key drug in this study, significantly improved survival rates compared with surgery alone in patients with rectal or colorectal cancer [13]. In patients with stage I lung adenocarcinoma, 2 years of UFT monotherapy revealed a significant impact on survival [17]. Moreover, most cases of recurrent colon cancer occur in the first 2 years after surgery [10]. Although the rate of recurrence reaches a peak between 1 and 2 years after surgery [11], adjuvant chemotherapy is discontinued within 6 months in many cases, and many recurrent lesions are found after discontinuation of adjuvant chemotherapy. It is estimated that continuing adjuvant chemotherapy for at least 1 year decreases recurrent events, delaying the timing of recurrence and thereby making the cumulative recurrence rate different from that in patients receiving 6-month treatment.

Since the 5-year DFS rate was 68.8% in the control group and 68.9% in the study group, statistical superiority was not verified. Although the recurrence and mortality rates at each time point in the study group were thought to be delayed compared with those in the control group, there were no differences in results, suggesting that treatment during the first 6 months determines whether and when recurrence occurs in the study group. Year-to-year changes in DFS in this study (supplementary Table S1, available at *Annals of Oncology* online) show that the rate of recurrence peaks between 1 and 2 years after surgery in both groups, irrespective of the duration of postoperative adjuvant chemotherapy.

Currently, 5-FU (5-FU/LV, capecitabine, UFT/LV, and S-1) and oxaliplatin are the only key drugs for postoperative adjuvant chemotherapy for colon cancer. The results of this study suggest that it is difficult to achieve better treatment results even if the same treatment is continued for a long time. Currently, short-course postoperative adjuvant chemotherapy is also being tested. The International Duration Evaluation of Adjuvant Chemotherapy is evaluating whether a 3-month adjuvant therapy is noninferior for the primary parameter to the 6-month identical therapy in patients with colon cancer. If shortened therapy could provide a noninferior treatment outcome, patients would be substantially relieved of the cumulative toxicity burden of oxaliplatin (e.g. peripheral neuropathy and allergic reactions).

In conclusion, this study, which compared 18 and 6 months of oral UFT/LV treatment, failed to verify the superiority of 18-month treatment over 6-month treatment in either DFS or OS. The important finding from this study is that not 18 months but 6 months of treatment is enough for postoperative UFT/LV for stage IIB/III colon cancer.

## funding

The study was funded and conducted by JFMC. The grant number is JFMC 33-0502. No commercial support was involved in the study.

## disclosure

YK has received honoraria from Chugai Pharmaceutical and Takeda Pharmaceutical. HB has received grants from Taiho Pharmaceutical and Japanese Foundation for Multidisciplinary Treatment of Cancer. CH has received grants and honoraria from Taiho Pharmaceutical. All remaining authors have declared no conflicts of interest.

## Supplementary Material

Supplementary Data
