# Supplementary material for: Randomized phase III trial of treatment duration for oral uracil and tegafur plus leucovorin as adjuvant chemotherapy for patients with stage IIB/III colon cancer: final results of JFMC33-0502
Source: Ann Oncol. 2015 Sep 7;26(11):2274–80. doi: 10.1093/annonc/mdv358 (PMC4621030; doi:10.1093/annonc/mdv358)
Supplement: Supplementary Data [file supp_26_11_2274__index.html]

Randomized phase III trial of treatment duration for oral uracil and tegafur plus leucovorin as adjuvant chemotherapy for patients with stage IIB/III colon cancer: final results of JFMC33-0502 — Randomized phase III trial of treatment duration for oral uracil and tegafur plus leucovorin as adjuvant chemotherapy for patients with stage IIB/III colon cancer: final results of JFMC33-0502 — Supplementary Data 

# Randomized phase III trial of treatment duration for oral uracil and tegafur plus leucovorin as adjuvant chemotherapy for patients with stage IIB/III colon cancer: final results of JFMC33-0502

## Supplementary Data

Supplementary Data

- Supplementary Data - Doc file
