# Supplementary material for: Randomized phase III trial of treatment duration for oral uracil and tegafur plus leucovorin as adjuvant chemotherapy for patients with stage IIB/III colon cancer: final results of JFMC33-0502
Source: Ann Oncol. 2015 Sep 7;26(11):2274–80. doi: 10.1093/annonc/mdv358 (PMC4621030; doi:10.1093/annonc/mdv358)
Supplement: Supplementary Data [file supp_mdv358_mdv358supp.doc]

Table S1. Disease-free survival (by year)

|  | |  |  |  |  |  |
| --- | --- | --- | --- | --- | --- | --- |
|  | Assigned treatment | 1st year | 2nd year | 3rd year | 4th year | 5th year |
|  | Group A: Standard treatment | 88 | 78.4 | 73.2 | 71.1 | 69.1 |
|  |  | ( 84.9 - 90.5 ) | ( 74.6 - 81.6 ) | ( 69.2 - 76.8 ) | ( 67.0 - 74.7 ) | ( 64.9 - 72.8 ) |
|  |  | 64 / 468 | 115 / 412 | 142 / 381 | 153 / 362 | 163 / 325 |
|  | Group B: Investigational treatment | 88.2 | 79 | 73.6 | 70.7 | 68.9 |
|  |  | ( 85.2 - 90.7 ) | ( 75.3 - 82.3 ) | ( 69.6 - 77.1 ) | ( 66.6 - 74.3 ) | ( 64.7 - 72.6 ) |
|  |  | 63 / 472 | 112 / 420 | 141 / 388 | 156 / 364 | 165 / 327 |
